# Biomarkers in EndoVascular Aneurysm Repair (EVAR) and Abdominal Aortic Aneurysm: Pathophysiology and Clinical Implications

**DOI:** 10.3390/diagnostics12010183

**Published:** 2022-01-13

**Authors:** Francesco Stilo, Vincenzo Catanese, Antonio Nenna, Nunzio Montelione, Francesco Alberto Codispoti, Emanuele Verghi, Teresa Gabellini, Mohamad Jawabra, Massimo Chello, Francesco Spinelli

**Affiliations:** 1 Department of Vascular Surgery, Campus Bio-Medico University, 00128 Rome, Italy; f.stilo@unicampus.it; 2 Department of Cardiovascular Surgery, Campus Bio-Medico University, 00128 Rome, Italy; a.nenna@unicampus.it (A.N.); n.montelione@unicampus.it (N.M.); f.codispoti@unicampus.it (F.A.C.); e.verghi@unicampus.it (E.V.); mohamad.jawabra@unicampus.it (M.J.); M.Chello@unicampus.it (M.C.); f.spinelli@unicampus.it (F.S.); 3 Residency Program of Vascular and Endovascular Surgery, University of Ferrara, 44121 Ferrara, Italy; teresa.gabellini91@gmail.com

**Keywords:** abdominal aortic aneurysm, endovascular aortic aneurysm repair, biomarkers, endoleak, matrix metalloproteinases, interleukin-6, cystatin C

## Abstract

Circulating biomarkers have been recently investigated among patients undergoing endovascular aortic aneurysm repair (EVAR) for abdominal aortic aneurysm (AAA). Considering the plethora of small descriptive studies reporting potential associations between biomarkers and clinical outcomes, this review aims to summarize the current literature considering both the treated disease (post EVAR) and the untreated disease (AAA before EVAR). All studies describing outcomes of tissue biomarkers in patients undergoing EVAR and in patients with AAA were included, and references were checked for additional sources. In the EVAR scenario, circulating interleukin-6 (IL-6) is a marker of inflammatory reaction which might predict postoperative morbidity; cystatin C is a promising early marker of post-procedural acute kidney injury; plasma matrix metalloproteinase-9 (MMP-9) concentration after 3 months from EVAR might help in detecting post-procedural endoleak. This review also summarizes the current gaps in knowledge and future direction of this field of research. Among markers used in patients with AAA, galectin and granzyme appear to be promising and should be carefully investigated even in the EVAR setting. Larger prospective trials are required to establish and evaluate prognostic models with highest values with these markers.

## 1. Introduction

Abdominal aortic aneurysm (AAA) is a multifactorial disease and a potentially life-threatening condition. Pharmacological approaches to slow aneurysm progression and limit the near-fatal risk of acute ruptures are currently under investigation to reduce the negative impact on the healthcare system [1]. Endovascular aortic aneurysm repair (EVAR) has now become the standard of care which has been shown to reduce morbidity and mortality [2]. However, short-term and long-term complications still hamper procedural success, and the most common complication is the residual perfusion of the aneurysmal sac (i.e., endoleak). Considering their frequency, patients undergo long-term surveillance screening with computed tomographic angiography or vascular ultrasound, which are limited by contrast administration or poor accuracy, respectively. Post-procedural complications and their assessment might impact the cost-effectiveness of EVAR.

Therefore, the study of circulating biomarkers has been progressively introduced in the medical literature and in the near future, these biomarkers might help in identifying conditions prone to develop complications, for both post-EVAR patients and patients with aortic aneurysm in follow up. This review will discuss the current biomarkers in the setting of AAA, with particular emphasis on patients undergoing EVAR.

## 2. Materials and Methods

An electronic database search through PubMed and Scopus was performed in October 2021. All studies describing outcomes of tissue biomarkers in patients undergoing EVAR were included, and references were checked for additional sources. Case reports, opinions, and editorials were excluded. Pre-clinical studies (non-human studies) were considered only for the last paragraph of the results.

## 3. Results

Pre-clinical evidence was considered only if directly connected with human results, and potential implications for future research are discussed separately. In the presentation of results, the treated disease (i.e., EVAR) and the untreated disease (i.e., AAA) will be separated.

## 4. Biomarkers and Compounds in EVAR (Treated Disease)

After AAA repair, the hormonal and metabolic stress-related inflammatory cascade, clinically referred to as “post-implantation syndrome”, is rapidly activated [3] by surgical trauma, ischemia–reperfusion injury and local cellular interactions [3]. Although endovascular repair reduces tissue manipulation compared with open surgery, intra-luminal manipulation of the thrombus using catheters is sufficient to initiate and sustain this strong systemic inflammatory response. Inflammatory cytokines and their regulatory activities have been extensively investigated in recent years, and a systematic review [3] concluded that IL-6 and IL-8 were particularly involved in the post-implantation syndrome and their role is greater in open surgery. IL-1b, IL-10, and TNF-a are other final common pathways of the post-implantation syndrome with no differences between open and EVAR techniques [3].

After EVAR, the most common and serious complication is endoleak, which is generally diagnosed with computed tomography during surveillance follow up or in case of symptoms. Assessment of markers released from the aneurysm wall into the bloodstream might potentially be an alternative for early endoleak detection [1]. Among those biomarkers, matrix metalloproteinases (MMPs) are soluble enzymes with lytic activity produced by macrophages and vascular smooth muscle cells. In particular, MMP-9 has been implicated in aneurysm growth through structural changes in the aortic wall and ECM remodeling, and its soluble release in the bloodstream has been advocated as a marker for endoleak, as the continuous perfusion of the aneurysm sac promotes MMP-9 release in the bloodstream [1]. A meta-analysis by Ng et al. [1] concluded that patients with endoleak have higher 3-month values of plasma MMP-9 levels compared to patients without endoleak (SMD 1.42, 95%CI 0.48-2.36, *p* < 0.003) [1], echoing previous studies [4].

Another feared complication of EVAR is perioperative renal dysfunction, related to contrast medium or reduced flow to renal arteries [2]. Anuria is a tardive event in acute kidney disease and urine output cannot be reliably considered the unique indicator. A recent systematic review [2] concluded that neutrophil gelatinase associated lipocalin (NGAL), cystatin C, and liver-type fatty acid binding protein (FABP-L) were the most promising for assessing postoperative renal failure after EVAR [2]. Considering “classic” biomarkers, serum creatinine >1.5 mg/dL remains a strong predictor of increased 30-day mortality (RR 3.0, 95% CI 2.3-4.1, *p* < 0.001) [5].

Another aspect recently reviewed in literature is the coagulation cascade. As aneurysm leads to increased thrombin generation and fibrin turnover, EVAR could produce similar results. A recent review [6] concluded that EVAR increased thrombin activation and fibrinolysis up to one year after procedure, suggesting that this period might be associated with increased risk of cardiovascular events and confirming previous findings [7]. Moreover, elevated levels of fibrinogen degradation products (FDP) were correlated with endoleak. However, the impact of these changes in the coagulation and fibrinolysis on the outcomes of EVAR should be still investigated.

**Figure 1:**
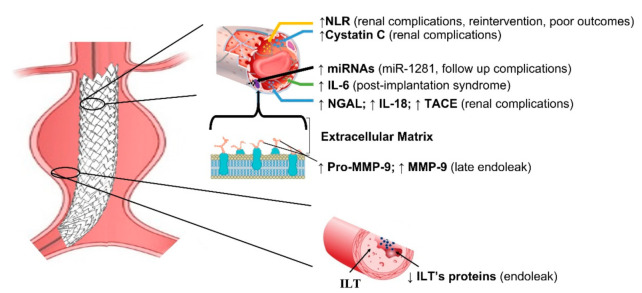
Schematic representation of main biomarkers in EVAR. NLR = Neutrophil–to–Lymphocyte ratio; IL-6 = interleukin 6; IL-18 = interleukin 18; NGAL = Neutrophil gelatin-associated lipocalin; TACE = tumor necrosis factor –α converting enzyme; ILT = intraluminal thrombus; ILT’s proteins = intraluminal thrombus proteins; MMP-9 = matrix metalloproteinase and tissue inhibitors; miRNAs = micro RNA.

Studies investigating biomarkers in EVAR disease are summarized in Table 1. Due to their genetic regulatory function and their high stability in biological fluids, plasmatic miRNAs have been highlighted as being optimal candidates as non-invasive biomarkers. For instance, a recent study found that elevated miRNA-1281 levels might identify patients with follow-up complications [8]. Most of the current literature is based on predicting post-operative renal disease, with Cystatin C being the recognized early marker of renal failure [9] (Figure 1).

## 5. Biomarkers and Compounds in Abdominal Aortic Aneurysms (Untreated Disease)

The thorough understanding of AAA pathophysiology has been clarified by proteomic analysis; specific proteins associated with AAA might be released from vascular tissue, intraluminal thrombus, tissue secretome, blood, and cells [10,11]. Proteomic analysis found biomarkers of complications, proteins related to pathogenic mechanisms, and potential therapeutic targets for AAA to be confirmed by tailored studies.

Differently from EVAR, in the setting of AAA there are known predictors of complications which have been investigated through the years such as serum elastin peptides (SEP) and plasmin–antiplasmin (PAP) complexes, MMP-9, IL-6, C-reactive protein (CRP), antitrypsin and IFN-gamma for AAA size, expansion rate or rupture [12,13,14,15,16]. Circulating, biomechanical, and genetic markers for AAA growth and rupture were reviewed in recent years [17,18,19,20,21,22].

**Figure 2:**
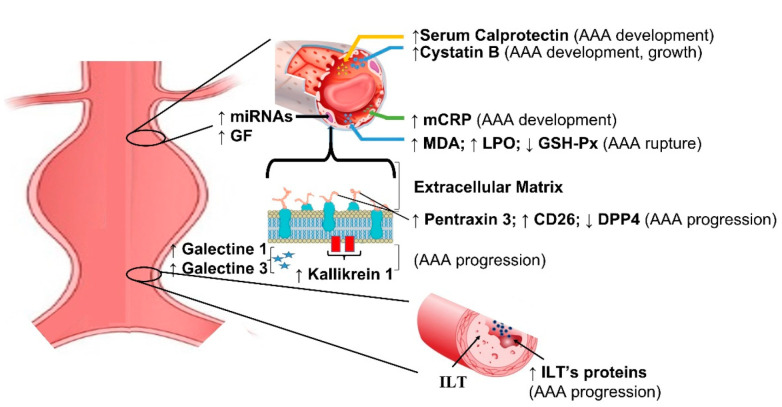
Schematic representation of main biomarkers in abdominal aortic aneurysms. mCRP = monomeric form of C-reactive protein; MDA = malondialdehyde; LPO = lipid hydroperoxide; GSH-Px = glutathione peroxidase; DPP4 = dipeptidyl peptidase–4; ILT = intraluminal thrombus; ILT’s proteins = intraluminal thrombus proteins; miRNAs = micro RNA; GF = genetic factors.

Current biomarkers in aortic aneurysm are summarized in Table 2. Genetic features of AAA have been extensively investigated in recent years using microarray, and genes involved in apoptosis, proteolysis, and humoral immune response yielded the most promising results. ALOX5, PTGIS, and CX3CL1 genes are potentially related with diagnosis of AAA [23,24]. Similarly, miRNA profiling produced under-expressed and overexpressed sequences suggesting the role in regulatory mechanisms [25,26,27]. Tissue factors related with inflammatory infiltrates and matrix degradation, such as pentraxin [28], galectin [29], calprotectin [30,31], kallikrein [32], and granzyme [33], correlate with the presence of AAA and might help in identifying patients at risk of AAA rupture (Figure 2).

Some recent reviews summarized the impact of miRNA and long non coding RNA (lncRNA) in cellular processed involved in abdominal aortic aneurysm [34,35,36,37,38,39]. Although some lncRNAs have been described as dysregulated in models or human tissue of AAA, comparatively few studies exist to date that have established their functional roles in pathologic disease.

## 6. The Common Background between EVAR and Untreated AAA: Extracellular Matrix

The pathogenesis of abdominal aortic aneurysm (AAA) is characterized by medial degeneration, manifesting with elastic fiber fragmentation, collagen fiber disorganization, and proteoglycan accumulation, as well as vascular smooth muscle cells (VSMC) loss [40]. The destruction of aortic connective tissue in AAA is led by a severe inflammatory reaction causing excessive degradation of the aortic extracellular matrix (ECM), which plays a pivotal role in AAA pathogenesis [40].

Vascular smooth muscle cells (VSMCs) play an important role in aorta homeostasis by secreting metalloproteinases (MMPs) and their inhibitors (TIMPs). As previously reported, MMPs, in particular MMP-9, have been implicated in aneurysm growth through structural changes in the aortic wall and ECM remodeling [40]. Previous studies have reported that excessive MMP secretion in the aortic wall leads to abnormal ECM degradation [41,42]. This important degradation of the ECM induces the release of cytokines which are involved in the regulation of ECM homeostasis [43]. All of these pathological events lead to weakening of the aortic wall, overexposing it to the biomechanical forces of pulsatile blood flow and blood pressure. Recent studies have reported a link between genetic defects in several collagen-encoding genes (i.e., COL1A1, COL1A2, COL3A1, COL5A1, and COL4A1/A2) and the development of AAA, thoracic abdominal aneurysm (TAA), and aortic dissection [44,45]. Moreover, mutation in COL3A1 can be found in more than 95% of patients with Ehlers-Danlos syndrome (EDS) with aortic complications [46]. An important role in the pathogenesis of AAA is represented by the degradation of elastin. The template for elastin is provided by fibrillin, which is a large extracellular glycoprotein that assembles to form microfibrils that are key components of most of the ECM. Fibrillin-1 (FBN1) and fibrillin-2 (FBN2) are the main structural components of the microfibril scaffold [45].

Pathologically increased of TGF-ß signaling was first implicated in the pathogenesis of TAA in the context of Marfan syndrome [47]. Furthermore, the role of dysregulated TGF- ß signaling in the pathogenesis of syndromic TAA is explained by the identification of loss-of-function mutations in TGF-ß receptors (TGFßR1 and TGFßR2), ligands (TGFß2 and TGFß3), and downstream effectors (SMAD2 and SMAD3) in patients with Loeys-Dietz syndrome [47,48]. Recently, new studies have proposed proteomics methods for a more systematic analysis of extracellular proteins [48,49]. One of the newest ECM proteomics approaches provides an activity-based proteomics method to relate the activity of specific proteases to ECM degradation components and to identify novel protease targets [49]. Furthermore, the latest glycoproteomics technologies allow an analysis of the glycosylation changes of ECM proteins in the arterial wall [50]. A recent finding, with a study on glycoproteomics, revealed an increase in MFAP4 in patients with MFS compared to control aneurysmatic patients [50]. Furthermore, TGF- ß was observed to induce MFAP4 expression in both human and mouse VSMCs, and MFAP4 was upregulated in aortic specimens from patients with a predisposition to AAA [50].

## 7. Evidence from Pre-Clinical Studies

Potential new biomarkers from recent pre-clinical studies are summarized in Table 3. Epigenetic mediators such as citrullinated histone H3 appear to be a promising therapeutic target for AAA [51]. Similarly, miRNA silencing and the PTEN pathway will have a role in modulating AAA progression and changing cell viability [52,53,54].

## 8. Future Directions

Despite the great interest and the flourishing literature in recent years, at present, the use of circulating biomarkers to detect complications of EVAR is still limited in clinical practice due to restricted availability and lack of recommendations from guidelines. To overcome those limitations, the methodology used to study the development and progression of aortic aneurysm should be translated into the EVAR scenario to support the use of biomarkers in the early diagnosis of complications. A potential first-in-clinic biomarker for EVAR would improve surveillance programs after hospital discharge, with tailored use of CT scan. Derivation and validation of a predictive model, according to age and sex, could be performed with a tailored registry analysis, with a synthetic evaluation of the most promising biomarkers such as IL-6, IL-18, cystatin C, MMP-9, and NGAL, similarly to point-of-care testing for platelet function.

An accurate analysis of the postoperative immune response will help in creating a more predictive biomarker panel for post-procedural morbidity. Future large prospective studies are required to identify the exact mechanisms of the cytokine interaction in the post-EVAR setting. Similarly, MMP-9 testing sensitivity and specificity should be evaluated in a real-life cohort before being considered as a surveillance test. Specific serum biomarkers could potentially form the basis of a tailored follow-up for patients with AAA or post-EVAR. Larger prospective trials are required to establish and evaluate prognostic models with highest values with these markers.

## 9. Conclusions

Circulating IL-6 is a marker of inflammatory reaction after EVAR and might act as a useful predictor of postoperative morbidity. Cystatin C is a promising early marker of post-procedural acute kidney injury after EVAR. Plasma MMP-9 concentration after three months from EVAR might help in detecting post-procedural endoleak. miRNAs are promising, but still limited in their clinical practice. Biomarkers for postoperative renal failure after EVAR are extremely debated in the literature, with some having strong references (NGAL, cystatin C) and others having weaker data to support their use (retinol binding protein, IL-18, N-acetyle-b-D-glocosaminidase).

In the setting of AAA, biomarkers have been more extensively investigated. Genetic factors and specific miRNA showed great association with AAA development and progression. Considering point-of-care testing, Galectin-1 and Galectin-3 might be extremely useful in clinical practice as biomarker of AAA progression, while Granzyme K and malondialdehyde are potential indicators of AAA rupture.

## Figures and Tables

**Table 1:** Biomarkers and compounds in EVAR (treated disease).

Biomarker/Compound	Biological Effect and Clinical Importance	Reference
Cystatin C	Early marker of renal failure–significant increase of Cystatin C 24 h after EVAR and for 12 months.	[9]
	Cystatin C levels in endovascular aneurysm repair patients significantly increased post- operatively and restored to values comparable to baseline at the discharge.	[55]
Platelet count and markers of platelet activation (CD-62P; CD36)	Significant reduction in PLT count and increase in PLT activation at the immediate postoperative period	[56,57]
Genetic features (MSN, PSMB10, and STIM1)	Three genes (MSN, PSMB10, and STIM1) are downregulated in AAA compared with controls; EIF3G, SIVA, PUF60, CYC1, FIBP, and CARD8 were downregulated post-EVAR. Those genes are involved in: regulation of apoptosis, proteolysis, the electron transport chain, leukocyte migration, and the humoral immune response	[24]
18F-fluorodeoxyglucose (FDG) detected with PET/CT; D-dimer	Patients who developed endoleak had a significantly higher SUVr compared to patients who did not develop endoleak. The SUVr was significantly higher in the group of patients with sac growth compared to patients with decreased AAA diameter. Quantitative analysis showed that sac growth and SUVr were significantly higher in the presence of endoleak after EVAR. D-dimer was significantly increased in patients with both endoleak and positive PET/CT in the post-EVAR group.	[58]
Matrix metalloproteinase and tissue inhibitors: MMP-2, MMP-9 (Pro and activate), TIMP-1, TIMP-2	Inguinal fascial tissue proMMP-9 significantly predicted late endoleak.	[59,60]
	ProMMP-9 and active MMP-9 biomarkers are significantly associated with late endoleak.	[61,62]
Osteopontin (OPN) and osteoprotegerin (OPG)	OPN (a calcification inhibitor) values are associated with aneurysm presence (more expressed in AAA group than in the control group, hernia pts); OPN values increase after AAA repair, independently of the type of repair.	[63]
Fibrinogen degradation product (FDP)	A change in FDP of 3.1 mg/mL was the optimal cutoff point for predicting the presence of an endoleak after EVAR	[64]
Albumin	Preoperative hypoalbuminemia is associated with increased postoperative morbidity and mortality in a severity-dependent manner among patients undergoing OAR or EVAR	[65]
	Preoperative hypoalbuminemia is associated with increased postoperative mortality in pts after FEVAR	[66]
Fibrinogen	the difference in fibrinogen levels (baseline to 24 h post-procedure) were significantly higher in patients with endoleaks	[6,67]
Neutrophil-to-lymphocyte ratio (NLR)	Preop NLR > 3 was independently associated with lower survival rates at 2-years	[68]
	NLR postoperative value of 9.9 PLR postoperative value of 22.8 were associated with the occurrence of AKI after EVAR	[69]
	High preoperative level of NLR (>3.6) was significantly associated with higher rates of death at 5 years as well as higher rates of reinterventions at 30 days, 1 year and 5 years.	[70]
Routine blood tests, white blood cell (WBC), C-reactive protein (CRP), procalcitonin (PCT)	WBC and CRP revealed that inflammatory markers were significantly enhanced as the volume of mural thrombus increased	[71,72]
	High C-reactive protein, high WBC and low postoperative procalcitonin are associated with post-implantation syndrome	[73]
miRNA	hyperexpression of miRNA-1281 in patients with AAA and a significant reduction of it after EVAR miRNA-1281 presents a significant reduction in patients with no follow-up complications	[8]
Urokinase plasminogen activator (suPAR), endothelin (ET)-1, tumour necrosis factor (TNF)-a, interleukin (IL)-6, IgM antibodies against phosphorylcholine (IgM anti-PC)	SuPAR (*p* < 0.001), ET-1 (*p* = 0.003) and IL-6 (*p* = 0.02) increased whereas IgM anti-PC decreased (*p* < 0.001) after EVAR	[74]
Neutrophil gelatin-associated lipocalin (NGAL), interleukin 18 (IL-18), and retinol-binding protein (urine levels)	A significant rise in levels of NGAL and IL-18 precedes the significant rise in Serum creatinine in pts with AKI after EVAR	[75,76]
Urinary liver-type fatty-acid-binding pro- tein (L-FABP)	A significant rise in level of urinary L-FABP precedes the rise of serum creatinine in pts with AKI after EVAR	[77,78]
High-mobility group box 1 (HMGB-1)	Serum HMGB-1: intracellular regulator of gene transcription and promotes secretion of several inflammatory cytokines. Serum HMGB-1 levels in AAA patients were significantly higher than in healthy controls; the serum HMGB-1 levels in both the EVAR group and the OAR group were significantly decreased from baseline at both 3 months and 1 year after surgery	[79]
Urinary Cystatin C (uCysC)	High postoperative level uCysC precedes the rise of serum creatinine in pts with AKI after EVAR or OR	[80]
Tumor necrosis factor-α converting enzyme (TACE), Notch-1	TACE and Notch1 concentrations were higher in patients with endoleak than in those without endoleak, 6 months after EVAR	[81]
Intraluminal thrombus (ILT)	Absence of ILT is a significant predictor of type II endoleak	[82]
Serum IL-1-α, IL-1β, IL-4, IL-6, IL-8, IL-10, IFN-γ, IP-10, MCP-1, TNF-α, and TNF-β	Significant decrease of IL-1α, 6 months after EVAR	[83]
Aspartate transaminase to platelet ratio index (APRI)	Significant increase in morbidity and mortality in pts with liver fibrosis after EVAR	[84,85]

**Table 2:** Biomarkers and compounds in thoracoabdominal aortic aneurysms (untreated disease).

Biomarker/Compound	Biological Effect and Clinical Importance	Reference
Genetic features	Three genes (MSN, PSMB10, and STIM1) are downregulated in AAA compared with controls. Those genes are involved in: regulation of apoptosis, proteolysis, the electron transport chain, leukocyte migration, and the humoral immune response.	[24]
	ALOX5, PTGIS, CX3CL1 genes are potentially related with diagnosis of AAA.	[23]
	Gene expression profiles allowed to select new potential cytometry markers: CNN1, MYH10, MYOCD, ENG, ICAM2, TEK.	[86]
	120 genes were differentially expressed in AAA. In particular genes associated with inflammatory responses and nuclear-transcribed mRNA catabolic process. The expression levels of IL6 correlated positively with RPL7A and negatively with RPL21. The expression of RPL21 and RPL7A was downregulated, whereas that of IL6 was upregulated in AAA.	[87]
miRNAs	miRNAs are small (19–24 nucleotides) and highly conserved non-coding RNAs involved in gene regulation; are involved in several processes, such as cellular differentiation, apoptosis, or tumorigenesis.	[23]
	miR-193b-3p, 125b-5p, 150-5p are potentially related with diagnosis of AAA hsa-miR-30a-GNG2 and hsa-miR-15b-ACSS2 interaction pairs may represent novel mechanisms for explaining the pathogenesis of AAA	[88,89,90]
	In AAA tissue, six miRNAs (miR-1, miR-27b-3p, miR-29b-3p, miR-133a-3p, miR-133b, and miR-195-5p) were underexpressed from 1.6 to 4.8 times and four miRNAs (miR-146a-5p, miR-21-5p, miR-144-3p, and miR-103a-3p) were overexpressed from 1.3 to 7.2 times, suggesting their involvement in a common regulatory mechanism	[25]
	A total of 31 miRNAs and 51 genes were selected as the most promising biomarkers of diagnosis of AAA.	[26,27]
Pentraxin 3 (PTX3)	PTX3 was upregulated in AAA and colocalized with inflammatory infiltrates.	[28]
C-reactive protein (CRP)-to-albumin ratio (CAR)	Increased serum CAR was found to be an independent predictor of the presence of AAA	[91]
Galectin-1	Gal-1 is highly induced and contributes to AAA by enhancing matrix degradation activity and inflammatory responses in experimental model; The pathological link between Gal-1 and AAA is also observed in human patients	[29]
Galectin-3	Gal-3 regulates chemotaxis and inflammation; has been reported as a prognostic marker for cardiovascular disease as it is linked to myocardial fibrosis, tissue remodeling, and heart failure development. Circulating Gal-3 levels were significantly correlated with aortic diameter in a concentration-dependent manner. Higher plasma Gal-3 concentrations may be a useful biomarker of AAA progression	[92]
Serum calprotectin	Serum calprotectin levels in AAA patients were three times higher than in healthy subjects	[30,31]
TLR2, TLR3, TLR4, and TLR9 single-nucleotide polymorphisms (SNPs)	TLRs are type I transmembrane proteins expressed on various immune cells, which recognize molecular patterns unique to pathogens or endogenous molecules released from dying or injured cells Heterozygous genotypes of the TLR2 2029C/T and TLR3 1377C/T and 27C/A SNPs may serve as genetic biomarkers of AAA incidence	[93]
Peroxiredoxins (PRDX)	PRDX are a ubiquitous family of thiol- specific antioxidant enzymes that control the levels of intracellular peroxide, which is involved in oxidative stress and signal transduction. PRDX2 plays a role as a negative regulator of the pathological process of AAA	[94]
Monomeric form of C-reactive protein (mCRP)	mCRP induces an inflammatory response by monocyte activation and reactive oxygen species formation to exacerbate tissue damage. AAA showed a characteristic deposition of mCRP, and multiple potentially pathologic signaling pathways were upregulated in AAA cases with strong CRP immunopositivity (pathways associated with atherosclerosis, acute phase response, complement system, immune system, and coagulation)	[95]
Proteins released by intramural thrombus (ILT)	3 proteins that are present in ILT, released by ILT and differs between fast and slow growth AAAs. Plasma Attractin correlates significantly with future AAA growth	[71,72]
Granzyme K (GZMK)	GZMK, a proinflammatory member of granzyme family, was first discovered in human lymphokine-activated killer cells, and mainly expressed by cytotoxic lymphocytes and monocyte/macrophage cells. infiltrated immune cells in AAA tissues and their associated marker genes: GZMK, CCL5, GZMA, CD2, EOMES, CD247, CD2, CD6, RASGRP1, and CD48 elevated GZMK expression both in serum and tissues is correlated with the presence of AAA, and serum GZMK may be a useful non-invasive marker that helps to identify AAA and its rupture risk	[20,33,96,97]
Low molecular weight metabolites	four amino acids (histidine, asparagine, leucine, isoleucine) and four phosphatidylcholines (PC.ae.C34.3, PC.aa.C34.2, PC.ae.C38.0, lysoPC.a.C18.2) were found to be significantly lower after adjustment for confounders among the AAA patients compared with the controls	[98]
Dipeptidyl peptidase-4 (DPP4)-inhibitors	Dipeptidyl peptidase-4 (DPP4 a.k.a. CD26) is a serine protease that exists as a membrane bound cell surface peptidase, and as a soluble form in the circulation. DPP4 gene expression is correlated with the expression of genes related to typical AAA processes and the protein was expressed by macro- phages, T-cells, B-cells and SMCs in aneurysm tissue	[99]
Long noncoding RNAs (lncRNAs)	lncRNAs have the potential to regulate the expression of genes at the epigenetic, transcriptional, and posttranscriptional levels and play an important role in physiological process. Microarray profile analysis and validation of significantly expressed lncRNA between patients with AAA and the control group	[100]
Cystatin B	Growth/differentiation factor 15 and cystatin B had the best ability to discriminate AAA from non-AAA. Higher baseline levels of myeloperoxidase were significantly associated with faster abdominal aortic aneurysm growth	[101]
Kallikrein-1	Serine protease that generates bradykinin, promoting inflammation. Kallikrein-1 blocking antibody reduced levels of cyclooxygenase-2 and secretion of prostaglandin E2 and active matrix metalloproteinase 2 and matrix metalloproteinase 9 from human AAA explants and vascular smooth muscle cells exposed to activated neutrophils	[32]
Serum lipid peroxidation products: malondialdehyde (MDA), lipid hydroperoxide (LPO), and glutathione peroxidase (GSH-Px)	the serum MDA and LPO among AAA cases were remarkably increased compared with those from the normal patients. Inversely, serum GSH-Px was significantly decreased in patients with AAA compared to the control group. Moreover, serum MDA level was significantly increased in cases with rupture AAA compared to those in selective AAA cases. Serum MDA may serve as the candidate biomarker for diagnosis of AAA and accurate identification of increased risks of AAA rupture.	[102]
Ankle brachial index (ABI)	Logistical regression analysis revealed a statistically significant negative association between initial monophasic posterior tibial artery waveform and abdominal aortic aneurysm presence in patients with ABI > 0.9.	[103]
Fibroblast growth factor 21 (FGF21)	FGF21 is a peptide hormone maintaining the homeostasis of glucose, lipid, and energy balance, which belongs to the human FGF superfamily that has crucial roles in a myriad of biological processes. FGF21 was statistically higher in patients with AAA than in control subjects. The protein levels of β-klotho (an essential co-receptor of FGF21) in abdominal aorta of AAA were found significantly lower than in control group.	[104]

**Table 3:** Potential new biomarkers from recent pre-clinical studies.

Biomarker/Compound	Study Type	Biological Effect and In-Vivo Implication	Reference
Neutrophil extracellular traps (NETs), citrullinated histone H3	mice	citH3 represents a promising AAA biomarker and potential therapeutic target. Inhibitor of citH3 block the AAA progression in mice.	[51]
miR-188-5p	mice	Expression of miR-188-5p is increased in experimental AAAs. Treatment with miR-188-5p inhibition limits experimental AAA progression, with histologic evidence of reduced neovessels and attenuated mural leukocyte infiltration.	[54]
myeloid related protein 8/14 (MRP8/14)	rat	MRPs especially for MRP8/14 increased the levels of MMP-2 and MMP-9 in rat models. RP8/14 was associated with AAA presence and progression	[105]
C1q/tumor necrosis factor (TNF)-related protein-13 (CTRP13)	mice	CTRP13 was shown to effectively reduce the incidence and severity of AAA in conjunction with reduced aortic macrophage infiltration, expression of proinflammatory cytokines (interleukin-6 [IL-6], TNF-α, and monocyte chemoattractant protein 1 [MCP-1]), and vascular smooth muscle cell (SMC) apoptosis. Mechanistically, nicotinamide phosphoribosyl-transferase 1 (NAMPT1) was identified as a new target of CTRP13. NAMPT1 knockdown blocked the beneficial effects of CTRP13 on vascular inflammation and SMC apoptosis. CTRP13 management may be an effective treatment for preventing AAA formation	[106]
Differentially expressed genes (DEGs) of proteases	mice	43 DEGs were correlated with the expression of the protease profile, and most were clustered in immune response module. Mmp16 and Mmp17 were significantly downregulated in AAA mice, while Ctsa, Ctsc, and Ctsw were upregulated. that these ectopic genes are potentially crucial to AAA formation and may act as biomarkers for the diagnosis of AAA.	[107]
microRNAs (miRNAs) miR-29a-3p, phosphatase and tensin homolog (PTEN)	mice	Increased expression of miRNA-29a-3p found in AAA-mimic cells with increased cellular viability and significant pathological apoptosis. Further, when the expression of miRNA-29a-3p was downregulated, it reduced the cell viability of AAA cells. PTEN was directly targeted by miRNA-29a-3p so as to regulate the AAA progression. Thus, PTEN was found to strengthen the proliferation effect of miRNA-29a-3p in AAA cells.	[52]

## Data Availability

PubMed: https://pubmed.ncbi.nlm.nih.gov/ (accessed on 1 November 2021); Scopus: https://www.scopus.com/home.uri (accessed on 1 November 2021).
